# Circular RNA ITCH suppresses proliferation, invasion, and glycolysis of ovarian cancer cells by up-regulating CDH1 via sponging miR-106a

**DOI:** 10.1186/s12935-020-01420-7

**Published:** 2020-07-23

**Authors:** Chunli Lin, Xiaofeng Xu, Qiumin Yang, Lu Liang, Shulin Qiao

**Affiliations:** 1 Department of Oncology, Shangqiu First People’s Hospital, No. 292, South Kaixuan Road, Shangqiu, 476100 Henan China; 2 Department of Obsterics, Zhengzhou Yihe Hospital, Zhengzhou, Henan China

**Keywords:** Ovarian cancer, circ-ITCH, miR-106a, CDH1, Invasion, Glycolysis

## Abstract

**Background:**

Accumulating data suggested that circular RNAs (circRNAs) played important roles in the development of human cancer. However, the potential mechanism of circRNAs in ovarian cancer remains unclear.

**Methods:**

Quantitative real-time polymerase chain reaction (qRT-PCR) was used to measure the levels of circRNA itchy E3 ubiquitin protein ligase (circ-ITCH), microRNA-106a (miR-106a) and E-cadherin (CDH1). Cell Counting Kit-8 (CCK-8) and Transwell assay were carried out to measure cell proliferation and invasion. Glucose consumption, lactate production, and ATP level were assessed by the glucose, lactate, and ATP assay kits, respectively. Cell apoptosis was detected by Flow cytometry. The binding sites were predicted by StarBase v.2.0 or microT-CDS and verified by dual-luciferase reporter assay and RNA immunoprecipitation (RIP) assays. CDH1 protein level was determined by western blot. The functional role of circ-ITCH was measured by xenograft tumor model in vivo*.*

**Results:**

Circ-ITCH was down-regulated in ovarian cancer and positively correlated with 5-year overall survival of patients with ovarian cancer. RNase R digestion assay confirmed that circ-ITCH was more stable than its linear mRNA form. Moreover, circ-ITCH was mainly distributed in the cytoplasm of ovarian cancer cells.

Functionally, circ-ITCH overexpression hindered proliferation, invasion, glycolysis and promoted apoptosis of ovarian cancer cells. Besides, circ-ITCH overexpression inhibited ovarian cancer cell progression by targeting miR-106a. Additionally, CDH1 was a target of miR-106a, and the protein level of CDH1 was negatively regulated by miR-106a. Similarly, CDH1 knockdown recovered the inhibition effects of miR-106a inhibitor or circ-ITCH overexpression on the progression of ovarian cancer cells. Importantly, circ-ITCH up-regulated the protein level of CDH1 by sponging miR-106a in ovarian cancer cells. Circ-ITCH overexpression suppressed the growth of ovarian cancer cells in vivo*.*

**Conclusion:**

Circ-ITCH suppressed proliferation, invasion, glycolysis, and promoted apoptosis of ovarian cancer cells by modulating the miR-106a/CDH1 axis.

## Background

Ovarian cancer is a highly malignant tumor in gynecological diseases and has a high mortality rate among women in the world [1, 2]. Despite the techniques of surgical treatment and drug chemotherapy have advanced, the overall survival rate of ovarian cancer patients remains low. Therefore, it is urgent to study new targets as diagnostic and prognostic markers.

Non-coding RNA (ncRNAs) has been shown to play pivotal roles in the occurrence and progression of many types of human tumors [3, 4]. Circular RNAs (circRNAs), a kind of ncRNAs, have covalently closed continuous ring structure and often regulate mRNA expression through competitive binding with microRNA (miRNA) [5, 6]. CircRNA has been reported to play a pivotal role in human cancer by participating in a variety of cell biological behaviors [7]. Others like Li et al. reported that circ-HIPK3 inhibited cell growth, angiogenesis, and lymph node metastasis in bladder cancer [8]. Chen et al. demonstrated that circRNA-100290 could promote glycolysis and cell proliferation in oral squamous cell carcinoma [9]. However, there are few studies on the function of circRNA in ovarian cancer.

CircRNA itchy E3 ubiquitin-protein ligase (circ-ITCH) has been widely reported to be down-regulated in osteosarcoma [10] and bladder cancer [11]. Guo et al. indicated that high expression of circ-ITCH was beneficial to the survival of hepatocellular carcinoma patients [12]. Besides, multiple studies indicated that circ-ITCH inhibited proliferation of ovarian carcinoma by down-regulating lncRNA HULC [13]. Circ-ITCH has also been reported to modulate cell proliferation and apoptosis by sponging miR-10a-α in epithelial ovarian cancer [14]. However, it is still unknown whether circ-ITCH can regulate tumor progression through other regulatory axes in ovarian cancer.

MiR-106a has been identified as an oncogene in many cancers, including ovarian cancer [15, 16]. MiR-106a overexpression could promote prostate cancer cell proliferation and metastasis [17]. Interestingly, miR-106a showed tumor inhibition effects on specific cancers, such as colorectal cancer [18], and miR-106a inhibited cell proliferation, invasion, and migration by targeting c-Jun in cervical cancer [19]. This expression pattern aroused our curiosity to investigate the function of miR-106a. As a cell adhesion transmembrane glycoprotein or tumor suppressor, the E-cadherin gene (CDH1) has been reported to be an important regulator of ovarian cancer [20]. It mainly regulated cancer cell metastasis by affecting cell adhesion ability [21]. However, whether CDH1 could be co-regulated by circRNA and miRNA to affect cell function in ovarian cancer remains further investigated.

In this report, we examined the expression of circ-ITCH in ovarian cancer tissues and cells and revealed a network of circ-ITCH/miR-106a/CDH1. The research on circ-ITCH was expected to provide a molecular marker for the diagnosis of ovarian cancer.

## Materials and methods

### Collection of tissue samples

The 45 ovarian cancer tissues and their pair-matched normal tissues were acquired from patients at Shangqiu First People’s Hospital from 2014 to 2018. This study was conducted after each patient completed an informed consent form. Each sample was immediately transferred to liquid nitrogen for freezing and stored at − 80 ℃. This study methodology was permitted by the ethics committee of Shangqiu First People’s Hospital. The clinical information of the patients including age, tumor size, FIGO stage, and Lymph node metastasis was summarized in Table 1.

**Table 1:** Clinicopathological variables and circ-ITCH level in ovarian cancer

Clinicopathologic features	Relative circ-ITCH level		*P* value
	Low (%)	High (%)	
Age (years)			0.7358
≥ 55	15 (60.0)	10 (40.0)	
< 55	11 (55.0)	9 (45.0)	
Tumor size (cm)			0.0009
< 4	9 (36.0)	16 (64.0)	
≥ 4	17 (85.0)	3 (15.0)	
Stage			0.0021
I-II	10 (38.5)	16 (61.5)	
III	16 (84.2)	3 (15.8)	
Lymph node metastasis			0.3803
No	13 (52.0)	12 (48.0)	
Yes	13 (65.0)	7 (35.0)	

### Cell culture and transfection

A2780 and OVCAR3 cell lines were purchased from Procell (Wuhan, China), and the ISOE80 cell line was obtained from the biotechnology company of Huzheng (Shanghai, China). All cells were cultured in Dulbecco’s modified eagle medium (DMEM, Thermo Fisher Scientific, Waltham, MA, USA) containing 10% fetal bovine serum (FBS, Hyclone, South Logan, UT, USA) and 0.1% penicillin/streptomycin (Thermo Fisher Scientific) at 37 ℃ with 5% CO_2_.

Overexpression vector of circ-ITCH was acquired by cloning the sequence of circ-ITCH into the pcDNA vector (RiboBio, Guangzhou, China). Small interfering RNAs against circ-ITCH (si-circ-ITCH) and CDH1 (si-CDH1) and their respective controls were synthesized by GenePharma (Shanghai, China). MiR-106a mimics, miR-control, miR-106a inhibitor, and anti-miR-control were synthesized by RiboBio. A2780 and OVCAR3 cells were transfected using Lipofectamine 2000 (Invitrogen, Carlsbad, CA, USA).

### Quantitative real-time polymerase chain reaction (qRT-PCR)

Trizol solution (Invitrogen) was employed to extract RNA from tissues and cells, and complementary DNA (cDNA) was synthesized by Prime Script RT Master Mix (Thermo Fisher Scientific). QRT-PCR for circ-ITCH, miR-106a, or CDH1 was performed on AB7300 thermo-recycler (Applied Biosystems, Foster City, CA, USA) using SYBR Select Master Mix (Applied Biosystems). Glyceraldehyde-3-phosphate dehydrogenase (GAPDH) was used as the internal parameter of circ-ITCH and CDH1, and U6 acted as an internal control for miR-106a. The sequences were listed as follows: circ-ITCH, forward: 5′-AGGATCCCAGGAGTTCAAAT-3′; reverse: 5′-GAGTGGGCTTGACTGAAATAG-3′. GAPDH, forward: 5′-TATGATGATATCAAGAGGGTAGT-3′; reverse: 5′-TGTATCCAAACTCATTGTCATAC-3′. CDH1, forward: 5′-ACACCATCCTCAGCCAAGA-3′; reverse: 5′-CGTAGGGAAACTCTCTCGGT-3′. MiR-106a, forward: 5′-GAGAACAGCAGGTCCAGCAT-3′; reverse: 5′-CTTCCT CAGCACAGACCGAG-3′. U6, forward: 5′-CTCGCTTCGGCAGCACA-3′; reverse: 5′-AACGCTTCACGAATTTGCGT-3′. The relative level was analyzed by the 2^−∆∆Ct^ method.

### RNase R treatment and subcellular localization

For RNase R treatment, total RNA (2 μg) was incubated at 37 ℃ with or without 3 U/μg of RNase R (Sigma-Aldrich, St. Louis, MO, USA). After treatment with RNase R, qRT-PCR was carried out to evaluate the expression levels of circular-ITCH and linear-ITCH.

For the nuclear and cytoplasmic fraction assay, a PARIS™ Kit (Invitrogen) was employed. Briefly, cells were harvested and lysed in cell fractionation buffer, followed by centrifugation to separate the nuclear and cytoplasmic fractions. The supernatant was transferred to a fresh RNase-free tube. The nuclear pellet was lysed in Cell Disruption Buffer. The cytoplasmic fraction and nuclear lysate were mixed with 2X Lysis/Binding Solution and then incubated with 100% ethanol. The RNAs of nuclear and cytoplasmic fractions were eluted with Elution Solution. U6 and GAPDH were employed as a positive control for nuclear and cytoplasmic fractions, respectively.

### Proliferation assay

The cell viability of A2780 and OVCAR3 cells was analyzed by Cell Counting Kit-8 (CCK-8) assay (Dojindo Japan). In short, transfected cells were seed in 96-well plates (2 × 10^3^ cells per well), followed by incubation for 24 h at 37 ℃. Afterward, 10 μL CCK-8 solution was added and cultured for another 4 h. Finally, a microplate reader (Bio-Rad, Hercules, CA, USA) at 450 nm was used to detect absorbance at various time points (0 h, 24 h, 48 h, and 72 h).

### Invasion assay

The invasion of A2780 and OVCAR3 cells was assessed by Transwell pre-coated with Matrigel (Invitrogen). Cells were suspended in 100 μL of serum-free medium and uniformly tiled to the top chamber of 24-well plates, and 600 μL of complete medium was added to the bottom chamber. After 24 h incubation, the cells attached to the bottom of the chamber were fixed and stained with 0.1% crystal violet (Psaitong, Beijing, China). Then, the migrated or invaded cells were counted under a microscope.

### Glucose consumption, lactate production, and ATP level assays

For detecting glucose consumption, A2780 and OVCAR3 cells were first transfected, and the supernatant of the cell medium was collected at 48 h after transfection. Then, the glucose concentration in the collected medium was assessed by a glucose assay kit (Sigma-Aldrich) according to the manufacturer’s instructions. Glucose consumption was calculated by measuring the glucose concentration in the original fresh medium minus the glucose concentration in the collected medium at the specified time.

Similarly, for measuring lactate production, the treated cells were harvested and the supernatant of the medium was extracted at the specified time. The concentration of lactate in the medium was measured by a lactate assay kit (BioVision, Milpitas, CA, USA). The optical density values at 570 nm were assessed with a SpectraMax® M2e Multimode Microplate Reader (Molecular Devices, LLC, Sunnyvale, CA, USA). Lactate production was calculated by subtracting the lactate concentration in the original fresh medium from the lactate concentration in the collected medium at the specified time.

Intracellular ATP assay kit (Biothema, Shanghai, China) was applied to detect ATP level according to the manufacturer’s instructions. Before cell analysis, the extracellular ATP was degraded by enzyme, and the ATP-degrading enzyme was deactivated at the same time. Finally, the ATP level was measured by Luciferase-based luminescent assay (Bio Thema AB, Handen, Sweden).

### Apoptosis assay

The apoptosis of A2780 and OVCAR3 cells was assessed by Flow cytometry using an Annexin V-fluorescein isothiocyanate (FITC)/propidium iodide (PI) kit (Keygen, Nanjing, China). The cells were first tiled into 6-well plates. After transfection for 48 h, the cells were harvested and suspended in Annexin V binding buffer. Then, the cells were double-stained using FITC and PI for 15 min without light, and cell apoptosis was examined by a BD FACS Calibur (Beckman Coulter, CA, USA).

### Dual-luciferase reporter assay

Wild type (circ-ITCH-WT or CDH1 3′UTR-WT) and mutant type (circ-ITCH-MUT or CDH1 3′UTR-MUT) containing miR-106a interacting sites or not were subcloned into the pmirGLO vector (YouBia, Changsha, China). 500 ng of dual-luciferase reporter plasmids and 2.5 µL of miR-106a mimics or miR-control were co-transfected to A2780 and OVCAR3 cells using Lipofectamine 2000 (Invitrogen) for 24 h. Finally, the luciferase activity was examined by a dual-luciferase reporter system (Promega, Madison, WI, USA). Each measurement was repeated at least three times.

### RNA immunoprecipitation (RIP) assay

A2780 and OVCAR3 cells were transfected with miR-106a mimics or miR-control, and RIP assay was performed by a Magna RIPTM RNA-binding protein immunoprecipitation kit (Millipore, Billerica, MA, USA). The cells were dissociated in lysis buffer after 48 h, and then the buffer containing magnetic beads conjugated with Ago2 or lgG antibody was added into cell lysates. The mixture was rotated overnight. The next day, immunoprecipitated RNA was separated after 30 min incubation with protease K. Finally, the levels of circ-ITCH and CDH1 were examined by qRT-PCR.

### Western blot assay

The treated A2780 and OVCAR3 cells were harvested, and proteins were extracted by RIPA (Beyotime, Shanghai, China). The concentration was measured by a Bicinchoninic acid (BCA) assay kit (Beyotime). Proteins were then fractionated and transferred to polyvinylidene difluoride (PVDF) membranes (GE Healthcare, Piscataway, NJ, USA). Next, the membranes were soaked in 5% milk powder for 2 h, and incubated with primary antibodies against CDH1 (1:1000, Cell Signaling Technology, Shanghai, China) or β-actin (1:2000, Abcam, Cambridge, MA, USA) overnight at 4 ℃. The membranes were incubated with horseradish peroxidase (HRP)-labeled secondary antibody (1:2000, Sigma-Aldrich) for 1 h. The bands were visualized using an ECL-PLUS kit (GE Healthcare, Piscataway, NJ, USA).

### In vivo experiment

This mice experiment was approved by the Animal Ethics Committee of the Shangqiu First People’s Hospital. A total of 15 five-week-old BALB/c nude mice (Shanghai Experimental Animal Center, Shanghai, China) were randomly divided into three groups. Then, OVCAR3 cells (1 × 10^7^) transfected with circ-ITCH, pcDNA, or Mock were subcutaneously inoculated into the flank of the nude mice. At 7 days after injection, tumor volume was detected once a week. All mice were sacrificed on day 28 after inoculation, and then the tumors were excised and weighed, followed by detection with RT-qPCR and western blot assays.

### Statistical analysis

Statistical analyses were conducted by Student’s *t*-test using SPSS22.0 software. The results were showed as mean ± standard deviation (SD) and repeated at least three times. The survival time of the two groups was compared by the Kaplan–Meier test. Differences were deemed to be statistically significant when *P* < 0.05.

## Results

### Circ-ITCH was down-regulated in ovarian cancer and correlated with poor prognosis

**Fig. 1:**
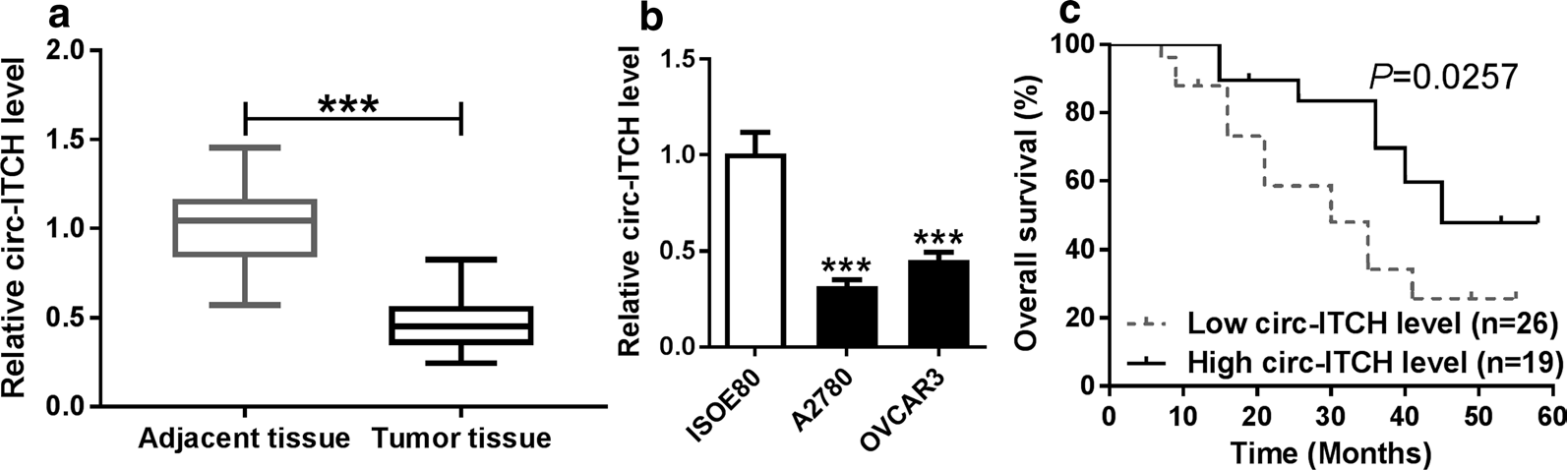
Circ-ITCH was down-regulated in ovarian cancer and correlated with poor prognosis. **a** The expression of circ-ITCH in 45 paired human ovarian cancer tissues and adjacent normal tissues was analyzed by qRT-PCR. **b** The expression of circ-ITCH in human ovarian epithelium cell line ISOE80 and ovarian cancer cell lines A2780 and OVCAR3 was detected by qRT-PCR. **c** The 5 year overall survival of ovarian cancer patients with high circ-ITCH level (n = 19) and low circ-ITCH level (n = 26) was analyzed by Kaplan–Meier survival analysis (*P* = 0.0257). ***P < 0.001

To explore whether the expression of circ-ITCH was changed in ovarian cancer, we performed qRT-PCR on 45 pairs of ovarian cancer tissues and adjacent normal tissues. The results showed that circ-ITCH was significantly repressed in ovarian cancer tissues compared with normal tissues (Fig. 1a). Meanwhile, circ-ITCH was found to be lower in human ovarian cancer cell lines A2780 and OVCAR3 compared with the ovarian epithelium cell line ISOE80 (Fig. 1b). Then, we analyzed the potential clinical significance of circ-ITCH expression in ovarian cancer patients. Patients were divided into two groups (High and Low) using the median value of circ-ITCH expression levels. As presented in Table 1, we found that low circ-ITCH expression was significantly associated with Tumor size (*p* = 0.0009) and FIGO stage (*p* = 0.0021). Additionally, ovarian cancer patients with low circ-ITCH level (n = 26) had a lower 5-year survival rate in comparison to those patients with high circ-ITCH level (n = 19) (*P* = 0.0257) (Fig. 1c). Our results suggested that low circ-ITCH expression led to poor prognosis in patients with ovarian cancer.

### Circ-ITCH overexpression inhibited proliferation, invasion, glycolysis, and promoted apoptosis of ovarian cancer cells

**Fig. 2:**
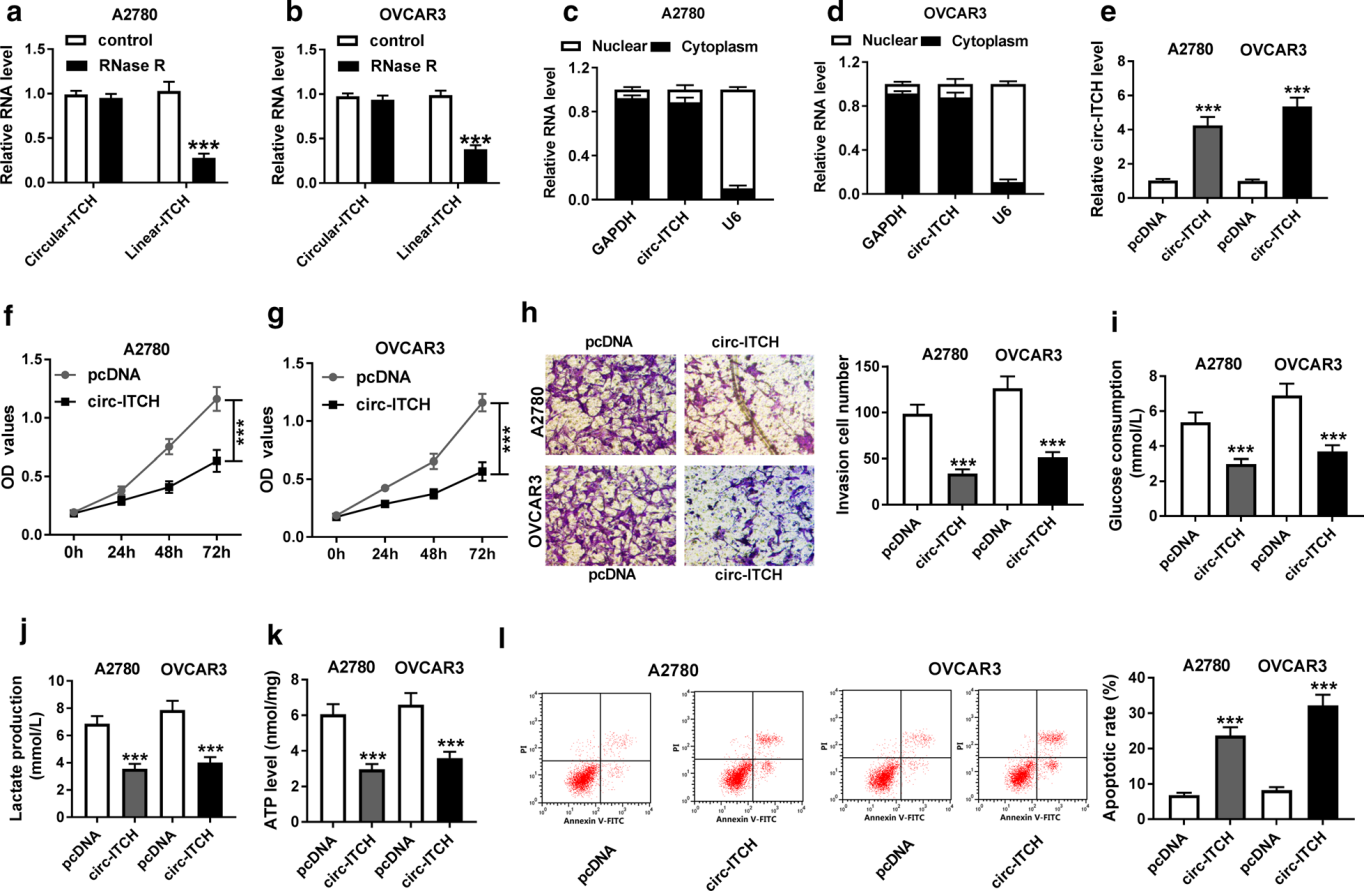
Circ-ITCH overexpression suppressed proliferation, invasion and glycolysis, and promoted cell apoptosis of ovarian cancer cells.** a**, **b** After RNase R treatment, linear-ITCH and circular-ITCH expression levels in A2780 and OVCAR3 cells were measured by qRT-PCR. **c**, **d** Relative expression of circ-ITCH in Nuclear and cytoplasm of A2780 and OVCAR3 cells was determined by qRT-PCR. **e** Circ-ITCH expression in A2780 and OVCAR3 cells transfected with pcDNA or circ-ITCH (pcDNA-circ-ITCH) was examined by qRT-PCR. **f**, **g** The proliferation ability of A2780 and OVCAR3 cells transfected with pcDNA or circ-ITCH was examined by CCK-8 assay. **h** The invasion of A2780 and OVCAR3 cells transfected with pcDNA or circ-ITCH was measured by Transwell assay with Matrigel. **i**–**k** Glucose consumption, lactate production and ATP level in A2780 and OVCAR3 cells transfected with pcDNA or circ-ITCH were detected using glucose, lactate and ATP assay kits, respectively. **l **The apoptosis of A2780 and OVCAR3 cells was detected by Flow cytometry. **P < 0.01, ***P < 0.001

To confirm the circular structure of circ-ITCH, RNase R treatment assay was performed in A2780 and OVCAR3 cells. As exhibited in Fig. 2a, b, the expression level of linear-ITCH was enormously decreased, while circular-ITCH was resistant to RNase R digestion. The results of qRT-PCR from nuclear and cytoplasmic fractions indicated that circ-ITCH was predominantly localized in the cytoplasm of A2780 and OVCAR3 cells (Fig. 2c, d). These data suggested that circ-ITCH harbored a loop structure and was mainly distributed in the cytoplasm of ovarian cancer cells. We then studied the functions of circ-ITCH by over-expressing circ-ITCH in A2780 and OVCAR3 cells. QRT-PCR assay indicated that circ-ITCH was markedly increased in A2780 and OVCAR3 cells transfected with over-expressed plasmid circ-ITCH compared to cells transfected with pcDNA (Fig. 2e). Functionally, overexpression of circ-ITCH decreased cell proliferation in A2780 and OVCAR3 cells (Fig. 2f, g). Moreover, Transwell assay showed that circ-ITCH overexpression notably reduced the invasion ability of A2780 and OVCAR3 cells (Fig. 2h). To evaluate the effect of circ-ITCH on glycolysis in ovarian cancer cells, we examined the relevant indicators of glycolysis in A2780 and OVCAR3 cells. The results showed that glucose consumption and lactate production were remarkably decreased after overexpression of circ-ITCH in A2780 and OVCAR3 cells (Fig. 2i, j). In addition, the level of ATP in A2780 and OVCAR3 cells were also significantly declined by circ-ITCH overexpression (Fig. 2k). Meanwhile, overexpression of circ-ITCH enormously increased the apoptosis rate (Fig. 2l). Overall, circ-ITCH played a vital regulatory role in the proliferation, invasion, glycolysis, and apoptosis of ovarian cancer cells.

### Circ-ITCH acted as a molecular sponge for miR-106a in ovarian cancer cells

**Fig. 3:**
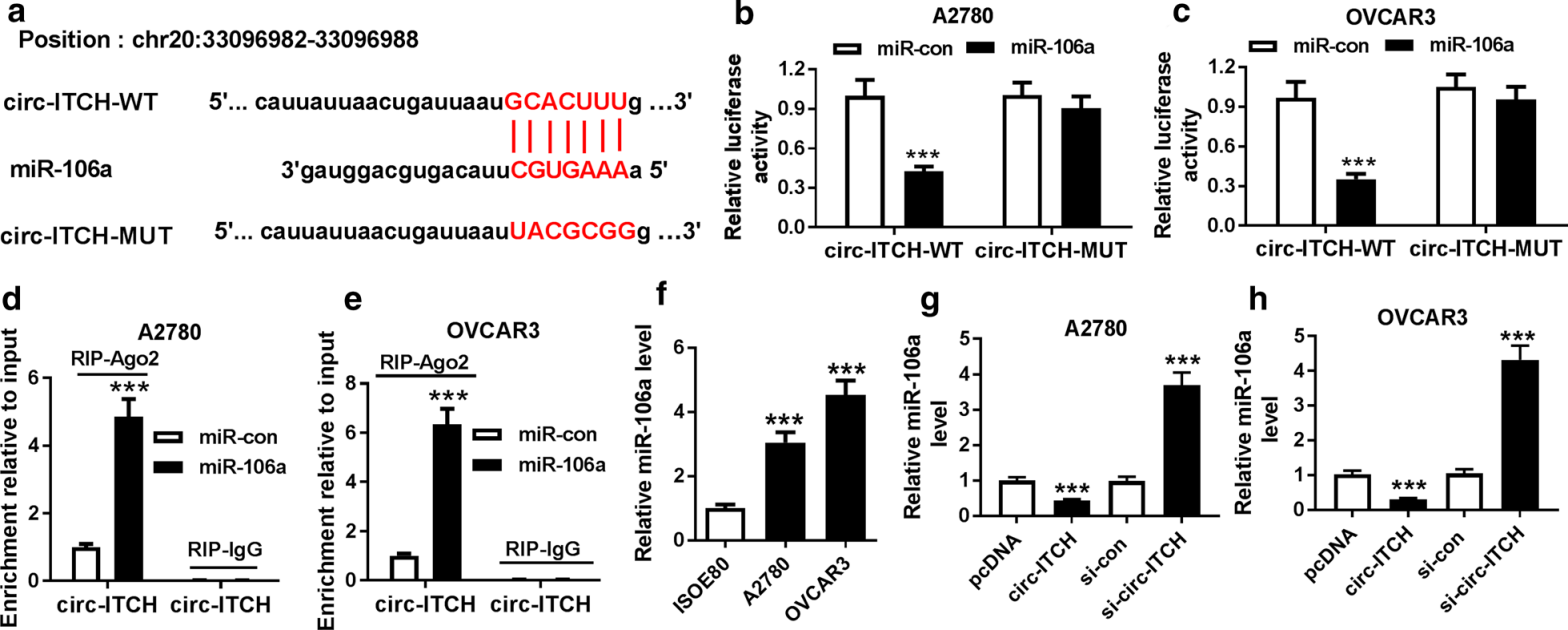
Circ-ITCH acted as a decoy for miR-106a in ovarian cancer cells.** a** The binding sites between miR-106a and circ-ITCH were predicted by StarBase v.2.0. **b**, **c** The relative luciferase activity in A2780 and OVCAR3 cells co-transfected with circ-ITCH-WT or circ-ITCH-MUT and miR-106a mimics (miR-106a) or miR-control (miR-con) was detected by dual-luciferase reporter assay. **d**, **e** Anti-Ago2 RIP assay was performed in A2780 and OVCAR3 cells after transfection with miR-106a or miR-con, and then the expression of circ-ITCH was examined by qRT-PCR. **f** MiR-106a expression in human ovarian epithelium cell line ISOE80 and ovarian cancer cell lines A2780 and OVCAR3 was detected by qRT-PCR. **g**, **h** The level of miR-106a in A2780 and OVCAR3 cells transfected with pcDNA, circ-ITCH, si-con (si-control) or si-circ-ITCH was examined by qRT-PCR. ***P < 0.001

CircRNAs function as competing endogenous RNAs (ceRNAs) to bind miRNAs and regulate mRNA expression [22]. We searched the downstream target genes of circ-ITCH by StarBase v.2.0. As shown in Fig. 3a, there were binding sites between circ-ITCH and miR-106a. Dual-luciferase reporter assay indicated that the luciferase activity in A2780 and OVCAR3 cells transfected with circ-ITCH-WT and miR-106a mimics (miR-106a) was significantly declined, and there was no significant change in the luciferase activity of cells transfected with circ-ITCH-MUT (Fig. 3b, c). To further confirm these results, we performed RNA immunoprecipitation (RIP) for Ago2 in A2780 and OVCAR3 cells. The data demonstrated that circ-ITCH was significantly enriched in the miR-106a group coated with the Ago2 antibody compared with the control group (Fig. 3d, e). Furthermore, the expression of miR-106a in ovarian cancer cell lines and ovarian epithelium cell lines was measured, and miR-106a was drastically increased in ovarian cancer cell lines A2780 and OVCAR3 (Fig. 3f). Moreover, qRT-PCR results showed that overexpression of circ-ITCH notably reduced the expression of miR-106a, and circ-ITCH knockdown significantly enhanced miR-106a expression in A2780 and OVCAR3 cells (Fig. 3g, h). These results supported that miR-106a was a target gene of circ-ITCH in ovarian cancer cells, and circ-ITCH negatively regulated the expression of miR-106a.

### MiR-106a reversed the inhibitory effect of circ-ITCH on ovarian cancer cell progression

**Fig. 4:**
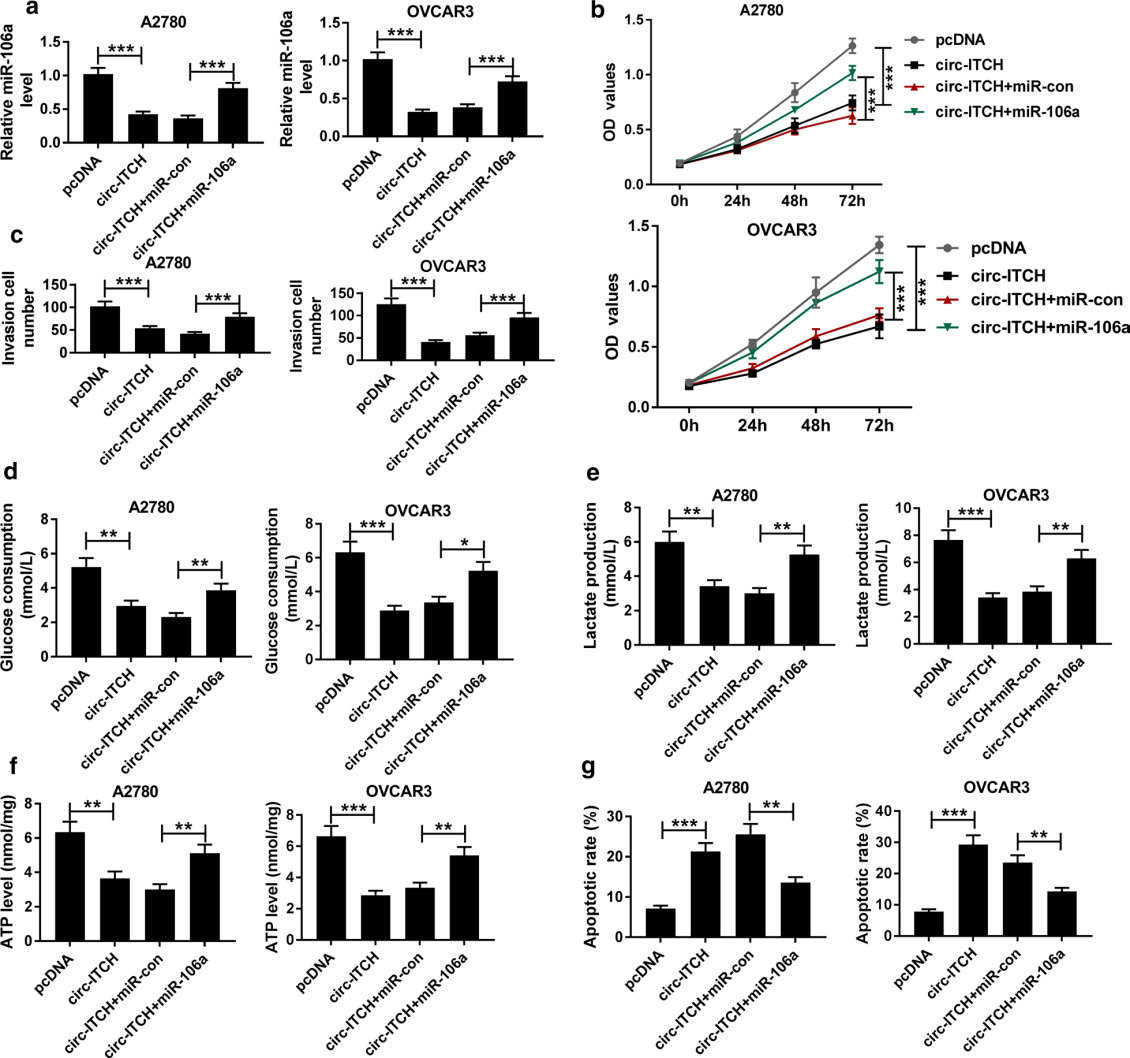
MiR-106a recovered the inhibitory effect of circ-ITCH on ovarian cancer cell progression.** a** The level of miR-106a in A2780 and OVCAR3 cells transfected with pcDNA, circ-ITCH, circ-ITCH + miR-con or si-circ-ITCH + miR-106a was measured by qRT-PCR. **b** The proliferation of A2780 and OVCAR3 cells was assessed by CCK-8 assay. **c** The invasion of A2780 and OVCAR3 cells was measured by Transwell assay with Matrigel. **d**, **f** Glucose consumption, lactate production and ATP level in A2780 and OVCAR3 cells were examined by glucose, lactate and ATP assay kits, respectively. **g** The apoptosis of A2780 and OVCAR3 cells was analyzed by Flow cytometry. *P < 0.05, **P < 0.01, ***P < 0.001

To investigate whether circ-ITCH exerted effects by sponging miR-106a, we conducted rescue experiments in ovarian cancer cells. Our results showed that overexpression of circ-ITCH in A2780 and OVCAR3 cells significantly repressed the expression of miR-106a, and this inhibition could overturn by miR-106a mimics (Fig. 4a). Moreover, miR-106a mimics recovered the inhibitory effects of circ-ITCH overexpression on proliferation (Fig. 4b), invasion (Fig. 4c), glucose consumption (Fig. 4d), lactate production (Fig. 4e) and ATP level (Fig. 4f) in A2780 and OVCAR3 cells. Then, we found that miR-106a mimics could also alleviate the promotion effect of circ-ITCH overexpression on the apoptosis rate of A2780 and OVCAR3 cells (Fig. 4g). In conclusion, circ-ITCH modulated the progression of ovarian cancer cells by sponging miR-106a.

### CDH1 was a direct target of miR-106a in ovarian cancer cells

**Fig. 5:**
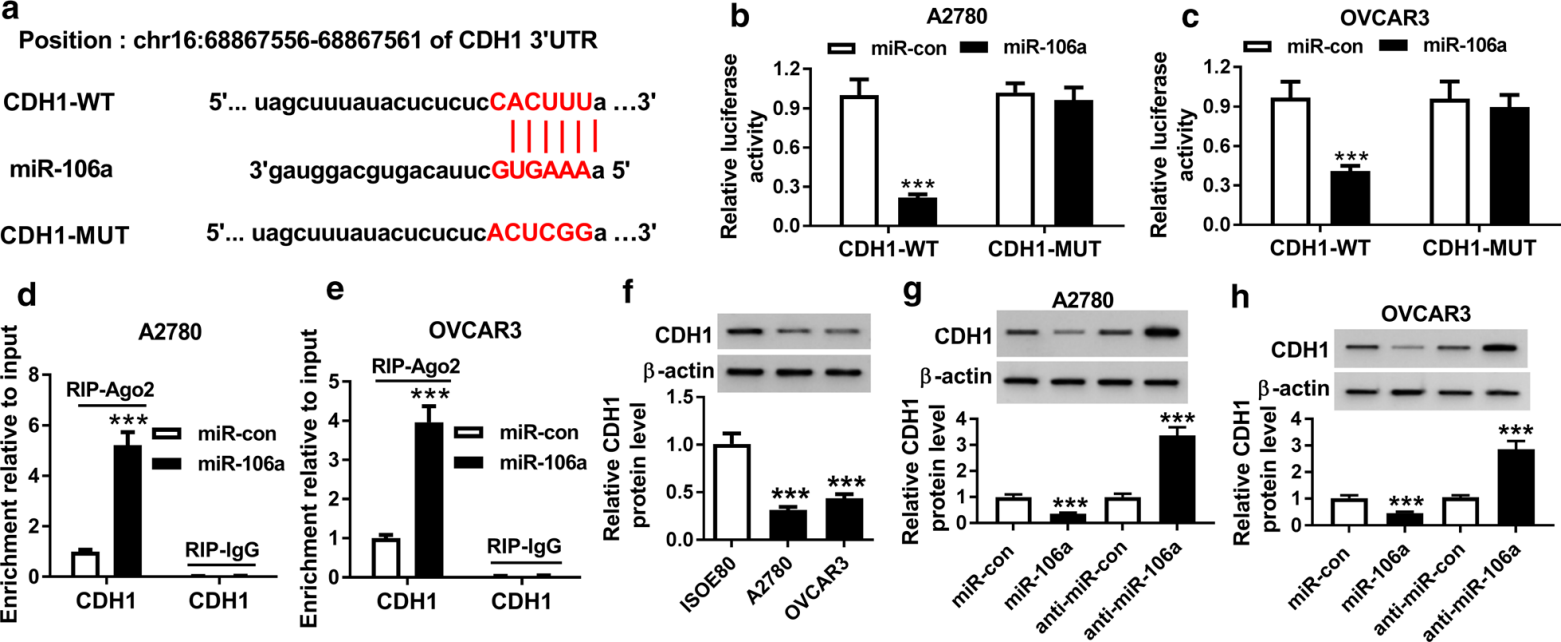
miR-106a directly targeted CDH1 in ovarian cancer cells. **a** The putative binding sites between miR-106a and 3′ UTR of LMO3 were predicted by microT-CDS. **b**, **c** The relative luciferase activity in A2780 and OVCAR3 cells co-transfected with CDH1-WT or CDH1-MUT and miR-106a or miR-con was assessed by dual-luciferase reporter assay. **d**, **e** CDH1 expression in A2780 and OVCAR3 cells transfected with miR-106a or miR-con and coated with Ago2 or IgG antibody was measured by qRT-PCR. **f** The protein level of CDH1 in human ovarian epithelium cell line ISOE80 and ovarian cancer cell lines A2780 and OVCAR3 was measured by western blot. **g**, **h** The protein level of CDH1 in A2780 and OVCAR3 cells transfected with miR-con, miR-106a, anti-miR-con or anti-miR-106a (miR-106a inhibitor) was examined by western blot. ***P < 0.001

We then predicted that there were binding sites between miR-106a and 3′ UTR region of CDH1 by microT-CDS (Fig. 5a). The results showed that overexpression of miR-106a in A2780 and OVCAR3 cells dramatically decreased the luciferase activity of CDH1-WT, while the luciferase activity of CDH1-MUT was not significantly changed (Fig. 5b, c). RIP assay indicated that CDH1 mainly enriched in miR-106a mimics (miR-106a) group incubated with the Ago2 antibody, implying the specific binding of miR-106a and CDH1 in A2780 and OVCAR3 cells (Fig. 5d, e). Subsequently, we examined the protein expression of CDH1 in ovarian cancer cell lines by western blot. We found that CDH1 protein expression was significantly retarded in ovarian cancer cell lines A2780 and OVCAR3 than that in normal cell line ISOE80 (Fig. 5f). Besides, the protein level of CDH1 in A2780 and OVCAR3 cells was significantly decreased by miR-106a and markedly increased by miR-106a inhibitor (anti-miR-106a) (Fig. 5g, h). These data implied that miR-106a directly targeted CDH1 and inversely modulated the protein expression of CDH1 in ovarian cancer cells.

### Silencing miR-106a inhibited cell progression in ovarian cancer by targeting CDHI

**Fig. 6:**
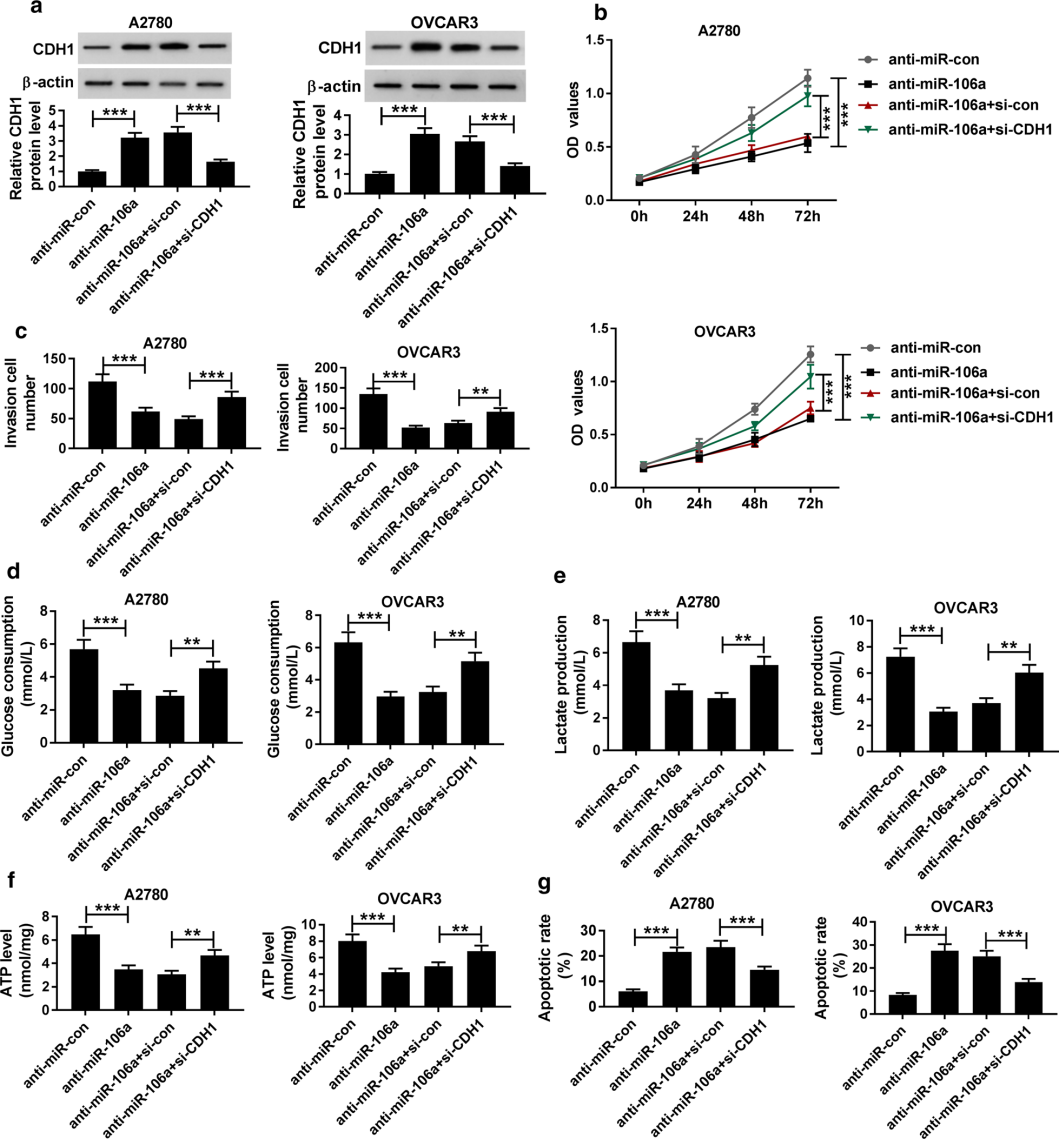
miR-106a knockdown inhibited cell progression in ovarian cancer by targeting CDHI. **a** The protein level of CDH1 in A2780 and OVCAR3 cells transfected with anti-miR-con, anti-miR-106a, anti-miR-106a + si-con or anti-miR-106a + si-CDH1 was measured by western blot. **b** The proliferation of A2780 and OVCAR3 cells was detected by CCK-8 assay. **c** The invasion of A2780 and OVCAR3 cells was assessed by Transwell assay with Matrigel. **d**–**f** Glucose consumption, lactate production and ATP level in A2780 and OVCAR3 cells were assessed using glucose, lactate and ATP assay kits, respectively. **g** The apoptosis of A2780 and OVCAR3 cells was analyzed by Flow cytometry. *P < 0.05, **P < 0.01, ***P < 0.001

Considering that miR-106a can regulate the expression of CDH1 in ovarian cancer cells, we further explored whether CDH1 could in turn affect the function of miR-106a on ovarian cancer cells. Western blot assay demonstrated that si-CDH1 inverted the promotion effect of miR-106a knockdown on CDH1 protein expression in A2780 and OVCAR3 cells (Fig. 6a). Functionally, si-CDH1 not only attenuated the impeditive impacts of interference with miR-106a in A2780 and OVCAR3 cells on proliferation (Fig. 6b), invasion (Fig. 6c), glucose consumption (Fig. 6d), lactate production (Fig. 6e) and the level of ATP (Fig. 6f) but also alleviated the promoting effect of miR-106a knockdown on apoptosis (Fig. 6g). These results revealed that miR-106a was involved in regulating the proliferation, invasion, glycolysis, and apoptosis of ovarian cancer cells by targeting CDH1.

### Circ-ITCH regulated CDH1 expression by targeting miR-106a

**Fig. 7:**
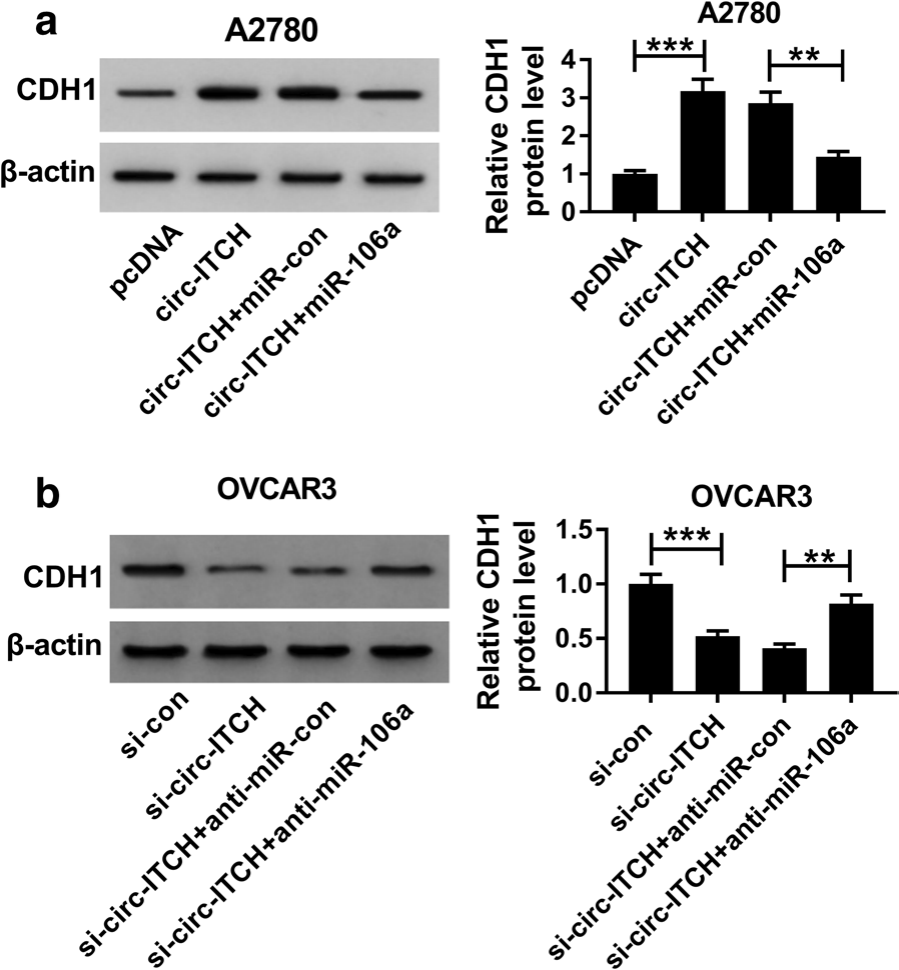
circ-ITCH regulated the expression of CDH1 by targeting miR-106a.** a** The protein level of CDH1 in A2780 and OVCAR3 cells transfected with pcDNA, circ-ITCH, circ-ITCH + miR-con or circ-ITCH + miR-106a was measured by western blot. **b** CDH1 protein level was detected in A2780 and OVCAR3 cells transfected with si-con, si-circ-ITCH, si-circ-ITCH + anti-miR-con, si-circ-ITCH + anti-miR-106a. **P < 0.01, ***P < 0.001

Given the targeting relationship between miR-106a and circ-ITCH or CDH1 in ovarian cancer cells, we further explored whether circ-ITCH could regulate CDH1 expression. Western blot results showed that overexpression of circ-ITCH enormously elevated the protein level of CDH1, while miR-106a overexpression partially offset the promoting effect and significantly reduced the protein level of CDH1 (Fig. 7a). Furthermore, miR-106a inhibition partially reversed the inhibitory effect of circ-ITCH depletion on the CDH1 protein level in A2780 and OVCAR3 cells (Fig. 7b). Overall, the results revealed that circ-ITCH could promote the expression of CDH1 by sponging miR-106a.

### Knockdown of CDHI partly abrogated the suppression effect of circ-ITCH on ovarian cancer cell progression

**Fig. 8:**
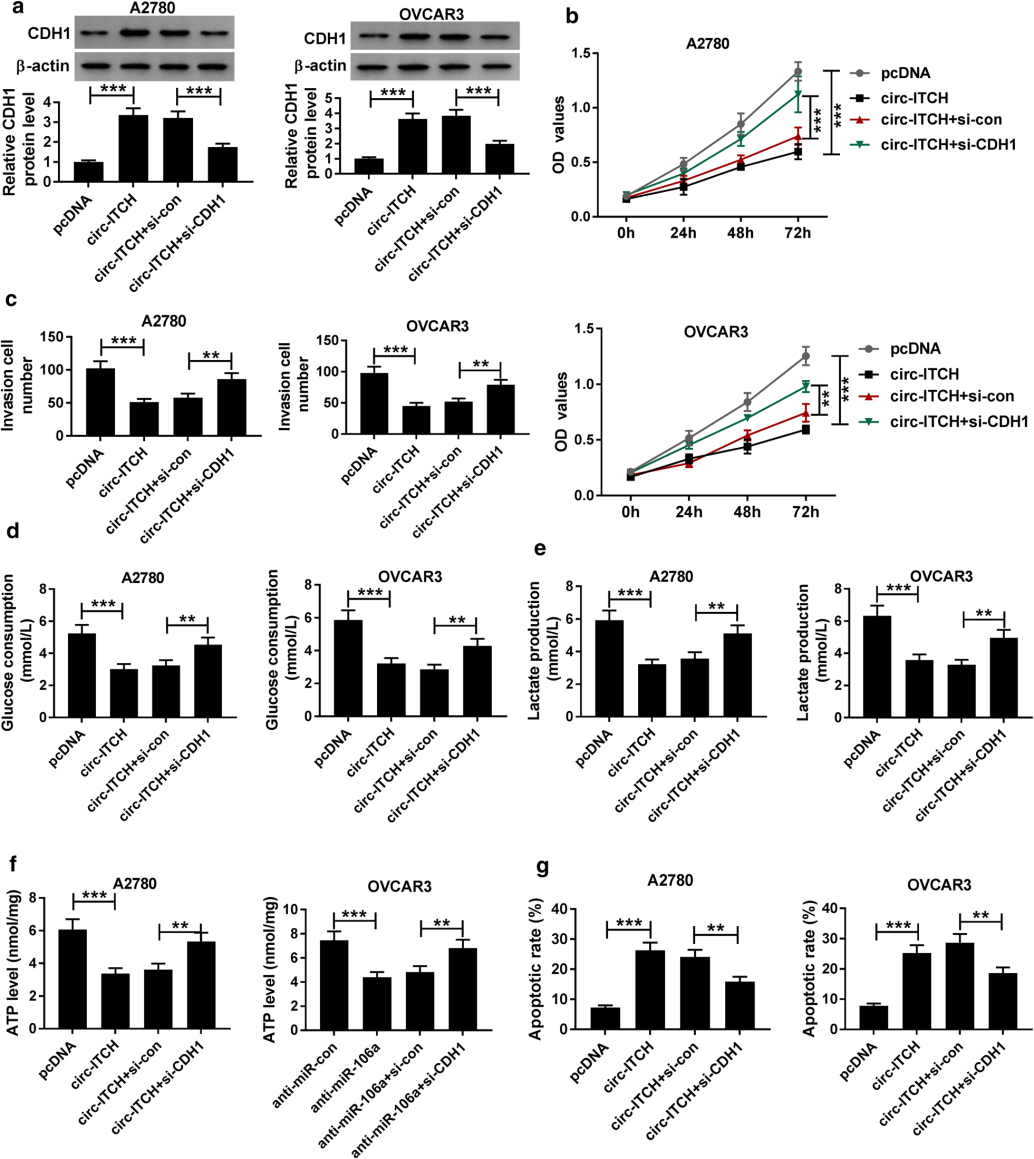
CDHI knockdown reversed the suppression action of circ-ITCH on ovarian cancer cell progression.** a** Western blot assay was applied to detect CDH1 protein level in A2780 and OVCAR3 cells transfected with pcDNA, circ-ITCH, circ-ITCH + si-con, or circ-ITCH + si-CDH1. **b** CCK-8 assay was performed to assess proliferation in transfected A2780 and OVCAR3 cells. **c** Transwell assay with Matrigel was used to measure invasion in A2780 and OVCAR3 cells. **d**, **f** Glucose, lactate and ATP assay kits were employed to examine glucose consumption, lactate production and ATP level in transfected A2780 and OVCAR3 cells, respectively. **g** Flow cytometry assay was conducted to assess apoptosis rate in transfected A2780 and OVCAR3 cells. **P < 0.01, ***P < 0.001

Next, we performed rescue assays to verify the regulatory effect of the circ-ITCH/miR-106a/CDH1 axis on ovarian cancer progression. As shown in Fig. 8a, overexpression of circ-ITCH facilitated the CDH1 protein level, while the silence of CDH1 significantly abolished the effect. Functional analysis suggested that upregulation of circ-ITCH resulted in an evident increase in proliferation (Fig. 8b), invasion (Fig. 8c), glucose consumption (Fig. 8d), lactate production (Fig. 8e), and ATP level (Fig. 8f), which was significantly eliminated by si-CDH1. Moreover, the promotion of cell apoptosis rate caused by circ-ITCH was abolished by CDH1 knockdown (Fig. 8g). All of these data indicated that circ-ITCH hindered the progression of ovarian cancer by miR-106a/ CDH1 axis.

### Circ-ITCH overexpression repressed the growth of ovarian cancer cells in vivo

**Fig. 9:**
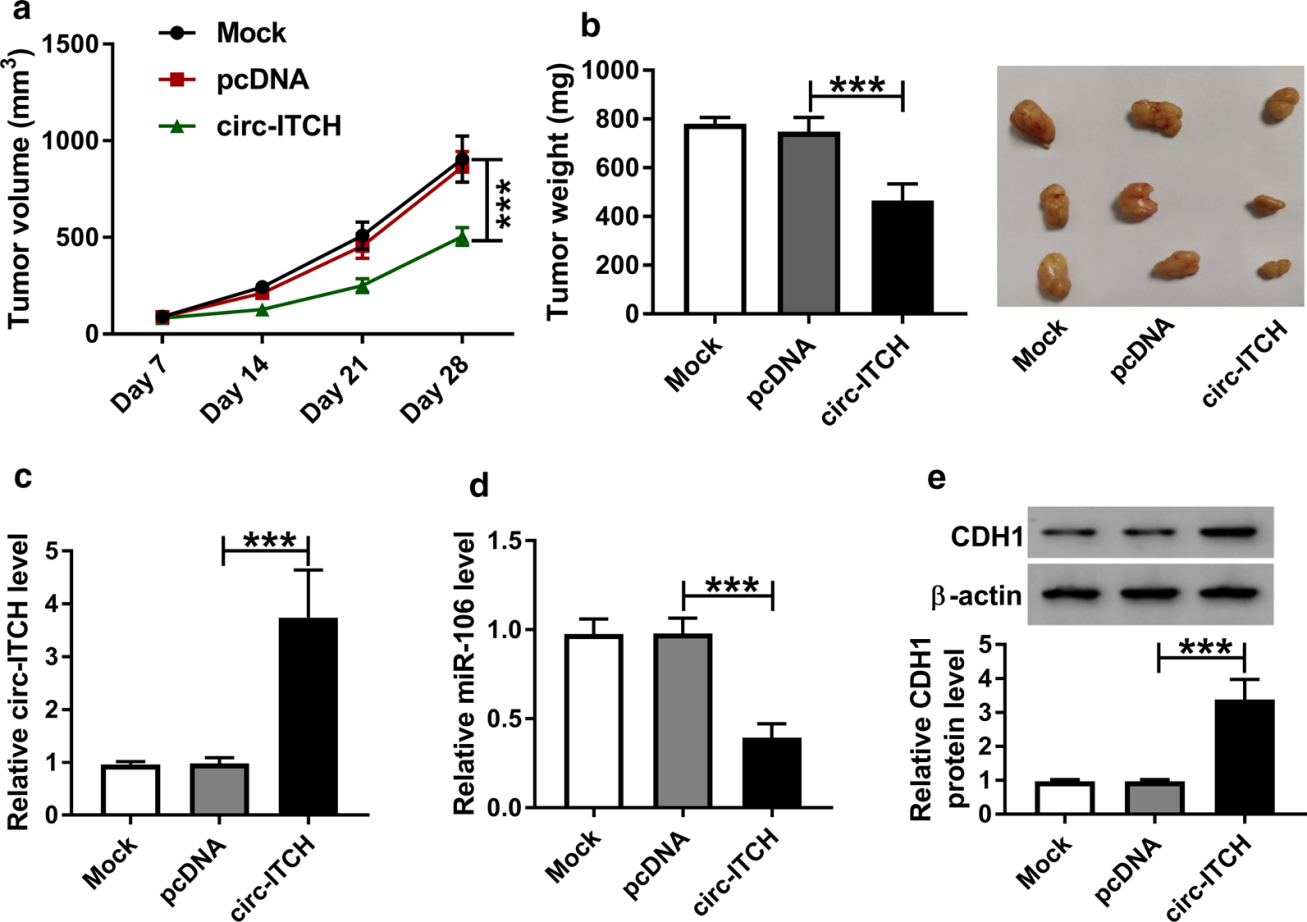
circ-ITCH upregulation suppressed the growth of ovarian cancer cells in vivo. **a**, **b** Tumor volume and tumor weight were detected in xenografts. **c**, **d** Expression levels of circ-ITCH and miR-106a were measured in xenografts by RT-qPCR assay. **e** CDH1 protein level was examined in xenografts by western blot assay. ***P < 0.001

In order to further verify the biological role of circ-ITCH in vivo, a xenograft tumor mouse model of ovarian cancer was establishment. Results suggested that tumor volume and weight were significantly reduced in circ-ITCH overexpression group (Fig. 9a, b)*.* Furthermore, RT-qPCR and western blot analysis indicated that the expression levels of circ-ITCH and CDH1 were increased, whereas the miR-106a level was decreased in tumor tissues in circ-ITCH-overexpression group (Fig. 9c–e). Together, these data suggested that upregulation of circ-ITCH could block the growth of ovarian cancer cells by regulating the miR-106a/CDH1 axis in vivo.

## Discussion

In recent years, increasing circRNAs have been discovered and their regulatory functions in human cancer had been confirmed [23]. However, the molecular mechanism of circRNA in ovarian cancer has not been thoroughly studied. Currently, the association between the expression of circ-ITCH and the ovarian cancer patient overall survival has been analyzed [24]. In this study, we found that circ-ITCH low expression was associated with the poor prognosis of the ovarian cancer patients. Our study also identified that circRNA circ-ITCH suppressed glycolysis, which was exceptionally constrained in ovarian cancer, and its low expression significantly reduced 5-year overall survival in ovarian cancer patients. The results were consistent with those of Luo et al. [14].

The invasion of cancer cells is the biggest obstacle in cancer treatment and increases the death rate of ovarian cancer patients [25, 26]. Moreover, numerous studies indicated that cancer cells rely on glycolysis for energy production, while normal cells rely on tricarboxylic acid (TCA) cycle for energy production [27]. The presence of glycolysis under aerobic conditions has been shown to be a "marker" of advanced cancer [28]. In this research, circ-ITCH enormously inhibited invasion and glycolysis of ovarian cancer cells and promoted cell apoptosis, suggesting that circ-ITCH was a tumor suppressor in ovarian cancer in vitro. Besides, the inhibitory effect of circ-ITCH overexpression on the growth of ovarian cancer cells was proved in nude mice. These results demonstrated that circ-ITCH exerted a tumor-suppressive role in ovarian cancer in vitro and in vivo*.* In agreement with our data, circ-ITCH was lowly expressed in ovarian cancer tissues and cells, and overexpression of circ-ITCH triggered the suppression effects on proliferation of ovarian cancer cells [13].

It has been widely reported that circRNAs, as ceRNAs of miRNAs, modulate the target genes of miRNAs [29]. For example, circRNA ITGA7 regulated colorectal cancer proliferation by sponging miR-3187-3p to elevate ASXL1 expression [30]. Thus, we speculated whether circ-ITCH could also play a role in ovarian cancer as a ceRNA. Firstly, we found that there were binding sites between circ-ITCH and miR-106a, and then a series of experiments proved that miR-106a could be directly targeted and negatively regulated by circ-ITCH. Moreover we found that miR-106a was remarkably upregulated in ovarian cancer cells. Subsequently, we over-expressed circ-ITCH and miR-106a simultaneously in ovarian cancer cells, and the results showed that miR-106a reversed the inhibition effects of circ-ITCH on cell invasion and glycolysis, and also attenuated the promotion effect of circ-ITCH on apoptosis. These results revealed that miR-106a was an oncogene in ovarian cancer, and circ-ITCH inhibited invasion, glycolysis and promoted apoptosis of ovarian cancer cells by targeting miR-106a. Our data were consistent with the results reported by Chen et al. [15].

CDH1, a cellular adhesive protein, plays a role in epithelial-mesenchymal transition (EMT) and is associated with tumor invasion and spread [31]. Moreover, CDH1 was confirmed to repress the levels of the matrix metalloproteinase 2 (MMP2) and MMP9 in Esophageal cancer [32]. The decreased expression of CDH1 could reduce the ability of cell adhesion, dissociation and inhibit the invasion of tumor [33]. Therefore, CDH1 is an invasion-inhibiting gene in most malignancies. In our study, miR-106a directly targeted CDH1 and inversely regulated its expression in ovarian cancer cells. In accordance with previous results [35], we demonstrated that CDH1 was notably down-regulated in ovarian cancer cells. Importantly, knockdown of CDH1 overturned the prohibitive impacts of silencing miR-106a on proliferation, invasion and glycolysis, and the promotion effect on apoptosis in ovarian cancer cells. Moreover, the results supported that circ-ITCH could up-modulate the level of CDH1 by sponging miR-106a in ovarian cancer cells. Taken together, circ-ITCH impeded cell proliferation, invasion and glycolysis by regulating the miR-106a/CDH1 axis, which was in agreement with previous reports that circ-ITCH retarded ovarian carcinoma progress by targeting the miR-145/RASA1 axis [35]. Furthermore, a circRNA has multiple binding sites of miRNAs, and a miRNA has thousands of target genes. In terms of circ-ITCH, there are many circ-ITCH-miRNA-mRNA networks. Thus, it is worth further exploring the mechanism of circ-ITCH in other cancers.

## Conclusion

In conclusion, we demonstrated that circ-ITCH served as a sponge of miR-106a to regulate CDH1 expression. Moreover, our data clarified that circ-ITCH repressed proliferation, invasion, glycolysis, and promoted apoptosis of ovarian cancer cells by targeting the miR-106a/CDH1 pathway. These results revealed the novel molecular basis of circ-ITCH in ovarian cancer progression.

## Data Availability

The datasets used and/or analyzed during the current study are available from the corresponding author on reasonable request.

## Abbreviations

circRNAs: circular RNAs
qRT-PCR: Quantitative real-time polymerase chain reaction
circ-ITCH: circRNA itchy E3 ubiquitin protein ligase
miR-106a: microRNA-106a
CDH1: E-cadherin
RIP: RNA immunoprecipitation
ncRNAs: Non-coding RNA; miRNA, microRNA
